# Imidazolium Salts for *Candida* spp. Antibiofilm High-Density Polyethylene-Based Biomaterials

**DOI:** 10.3390/polym15051259

**Published:** 2023-03-01

**Authors:** Clarissa Martins Leal Schrekker, Yuri Clemente Andrade Sokolovicz, Maria Grazia Raucci, Claudio Alberto Martins Leal, Luigi Ambrosio, Mário Lettieri Teixeira, Alexandre Meneghello Fuentefria, Henri Stephan Schrekker

**Affiliations:** 1 Institute of Basic Health Sciences, Universidade Federal do Rio Grande do Sul (UFRGS), Rua Sarmento Leite 500, Porto Alegre 90050-170, RS, Brazil; 2 Laboratory of Technological Processes and Catalysis, Institute of Chemistry, Universidade Federal do Rio Grande do Sul (UFRGS), Avenida Bento Gonçalves 9500, Porto Alegre 91501-970, RS, Brazil; 3 Institute of Polymers, Composites and Biomaterials, National Research Council of Italy (IPCB-CNR), Viale John Fitzgerald Kennedy 54, Mostra d’Oltremare Padiglione 20, 80125 Naples, Italy; 4 Laboratory of Biochemistry and Toxicology, Instituto Federal Catarinense (IFC), Rodovia SC 283—km 17, Concórdia 89703-720, SC, Brazil; 5 Faculty of Pharmacy, Universidade Federal do Rio Grande do Sul (UFRGS), Avenida Ipiranga 2752, Porto Alegre 90610-000, RS, Brazil

**Keywords:** ionic liquid, human mesenchymal stem cells, biocompatibility, melt blending, histopathological evaluation

## Abstract

The species of *Candida* present good capability to form fungal biofilms on polymeric surfaces and are related to several human diseases since many of the employed medical devices are designed using polymers, especially high-density polyethylene (HDPE). Herein, HDPE films containing 0; 0.125; 0.250 or 0.500 wt% of 1-hexadecyl-3-methylimidazolium chloride (C_16_MImCl) or its analog 1-hexadecyl-3-methylimidazolium methanesulfonate (C_16_MImMeS) were obtained by melt blending and posteriorly mechanically pressurized into films. This approach resulted in more flexible and less brittle films, which impeded the *Candida albicans, C. parapsilosis*, and *C. tropicalis* biofilm formation on their surfaces. The employed imidazolium salt (IS) concentrations did not present any significant cytotoxic effect, and the good cell adhesion/proliferation of human mesenchymal stem cells on the HDPE-IS films indicated good biocompatibility. These outcomes combined with the absence of microscopic lesions in pig skin after contact with HDPE-IS films demonstrated their potential as biomaterials for the development of effective medical device tools that reduce the risk of fungal infections.

## 1. Introduction

Nowadays, polymer-based medical devices such as catheters [1,2,3], prostheses [1,2], endotracheal tubes [1,2], implants [1,2], tissues for tissue engineering [1], drug delivery systems [3] and heart valves [1], are commonly used in hospitals. *Candida* spp. (e.g., *C. tropicalis*, *C. albicans* and *C. parapsilosis*) have a strong tendency to colonize these polymeric surfaces, forming fungal biofilms [4,5]. By definition, biofilms are complex communities of microorganisms, with a high degree of organization, characterized by cells that are adhered to a surface or interface and embedded in an extracellular matrix of extrapolymeric substances (polysaccharides, proteins, lipids and DNA) of microbial origin, producing a spatially organized three-dimensional structure [6]. Chemical communication between cells, called quorum sensing, allows microorganisms (bacteria and fungi) to coordinate their activity and group together in communities that provide similar benefits as those of multicellular organisms [1,7]. The process of biofilm formation occurs through adhesion to medical devices, which is arbitrated by the proteins of the cell wall. As biofilms are highly adherent, the in vivo destruction of the biofilm requires the removal of the contaminated medical device, and this procedure could result in medical complications [6,7,8]. Altogether, the *Candida* sp. biofilms elevate the probability of nosocomial infections in immunocompromised patients due to therapeutic failure and the elevated resistance to important antifungal drugs [9], such as amphotericin B and azoles [1,10,11].

Within this context, the development of biomaterials with improved antibiofilm properties is highly desired. As such, the utilization of high-density polyethylene (HDPE) with imidazolium salt (IS) additives is promising for the development of biomaterials for use in medical devices. This polymer has excellent mechanical and biological properties, turning this material extremely suitable for applications in medical devices [12], which has been explored in facial implants as a substitute for the human skeleton in bone regeneration [13,14], tangible bone implants [12], tissue engineering (scaffolds) [15], reconstruction of nasal cartilage [16] and catheters [17,18].

The application of drug additives in HDPE-based medical devices represents an emerging technology. Interestingly, these devices do not have the primary purpose to act as drug reservoirs but may contain the latter, leading to an adjunctive pharmacological action. The incorporation of additives, e.g., antibiotics into temporary or permanent implants is being used in an attempt to reduce infections and to improve the acceptance of organic implanted material, minimizing the possibility of rejection [17,18].

Imidazolium salts (ISs) have an ion that is a cationic version of a neutral imidazole heterocycle and are known for presenting various advanced properties [19,20,21]. When ISs are in the liquid state at 100 °C, these are classified as ionic liquids. In general, ISs are attractive substances for various chemical and pharmaceutical applications, principally due to their thermal and chemical stability, neglectable volatility, and modifiable physical and chemical properties through structural modifications [22,23,24,25]. Various biological activities and applications of ISs have been identified, such as antibacterial [24,25,26], antifungal [26,27,28], antitumor [24], antioxidant [24], antifibrous [24], bioengineering [24,26] and anti-inflammatory [29].

Currently, there are few truly effective antifungal drugs against emerging yeasts. A variety of *N*-alkyl-substituted ISs was screened in vitro to verify the antifungal activity against *C. glabrata*, *C. parapsilosis*, *C. tropicalis*, and *Trichosporon asahii*. The best activity against fungal growth was determined for the ISs 1-hexadecyl-3-methylimidazolium chloride (C_16_MImCl) and 1-hexadecyl-3-methylimidazolium methanesulfonate (C_16_MImMeS). This, in combination with the absence of cytotoxicity and damage to human leukocytes, turns these substances into promising drug leads [23]. Interestingly, the pre-treatment of catheter surfaces with C_16_MImCl impeded the growth of *C. tropicalis* biofilms [30]. Complementing this preventive action and in comparison to chlorhexidine, the gold standard for asepsis in hospitals, much lower concentrations of C_16_MImCl and C_16_MImMeS were necessary to effectively remove *C. tropicalis* biofilms on polystyrene microtiter surfaces [31]. In addition to these ISs, *t*-BuOH-functionalized ISs with varying *N*-alkyl chain lengths were studied for their antimicrobial and antibiofilm properties. The one with the longest *N*-alkyl chain, dodecyl, was the most effective to inhibit the biofilm growth of *Staphylococcus epidermidis* and *C. albicans* [32].

**Figure 1:**
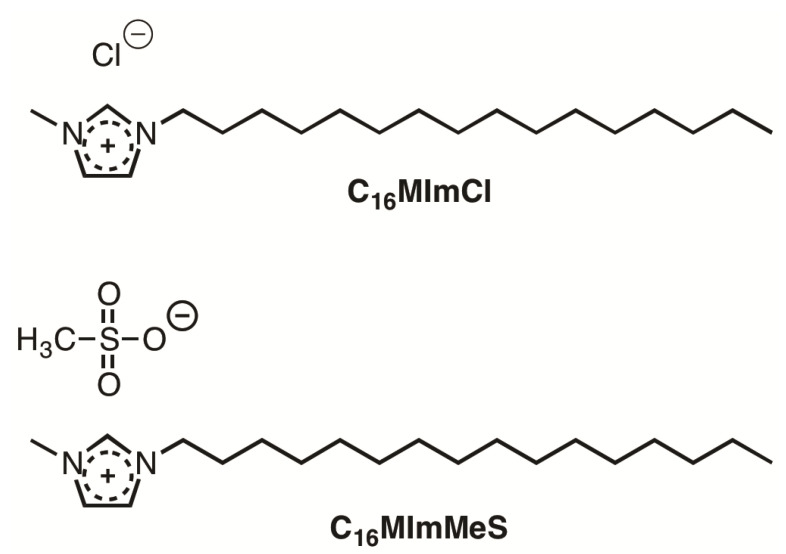
ISs C_16_MImCl and C_16_MImMeS applied in this study.

Poly(L-lactide) films containing an IS additive (C_16_MImCl or C_16_MImMeS) have been prepared by solvent casting which presented effective antibiofilm activities against *C. albicans*, *C. parapsilosis* and *C. tropicalis* [33]. The above-mentioned materials are biocompatible, do not cause skin irritation, and retain the original poly(L-lactide)’s mechanical and thermal properties. The incorporation of IS additives in polymers is an encouraging route to obtain biomaterials. Herein, HDPE-based biomaterials were obtained through melt-blending with an IS (C_16_MImCl or C_16_MImMeS) (Figure 1). The resulting biomaterials were characterized, including their antibiofilm properties against *Candida* spp.

## 2. Materials and Methods

**Materials.** HDPE (HA 7260, Braskem S.A., Triunfo, RS, Brazil) and C_16_MImCl (CJC China Jie Chemical, Shanghai, China) were donated and purchased, respectively. C_16_MImMeS was prepared using the synthesis reported [20]. For the removal of residual water, HDPE and the ISs were vacuum dried at 60 °C for 5 h.

**Yeast Strains.** The following phenotypically identified biofilm-forming yeast strains were selected: *C. albicans* CA04; *C. parapsilosis* RL11, RL20 and *C. tropicalis* ATCC750, ATCC950, 17A, 57A, 72A, 102A, 17P, 72P, 94P, RL15, RL16, RL17. These isolates belonged to the mycology collection of the Laboratory of Applied Mycology at UFRGS [33].

**Melt-Blended HDPE-IS and Film Formation.** HDPE was melt-blended without or with an IS (0, 0.125, 0.250, or 0.500 wt%), using a twin-screw extruder (HAAKE Rheomex PTW 16 OS, Thermo Fisher Scientific, Waltham, MA, USA). The IS was added after 1 min to the molten polymer, and the components were mixed for 6 min, maintaining the screw speed and temperature of 60 rpm and 190 °C, respectively. Processed samples were left cooling to 25 °C, air dried and milled. After vacuum drying at 60 °C for 5 h, the samples were pressed into 0.5 mm thick films using a hydraulic press (Monarch 3710, Carver, Wabash, IN, USA). Initially, the material was molten within 4 min at 190 °C, and then pressed for 30 s at 4 lbf. The obtained films were abbreviated as HDPE.IS.content (e.g., HDPE.MeS.0125 for HDPE containing 0.125 wt% of C_16_MImMeS).

**Scanning Electron Microscopy (SEM).** A scanning electron microscope (EVO 50, Carl Zeiss AG, Oberkochen, Germany) was used to study: (A) the morphology of the HDPE and HDPE-IS film surfaces and (B) the biofilm inhibition on these films through reported protocols [33].

**Atomic Force Microscopy (AFM).** The surfaces of the HDPE and HDPE-IS films were studied with the aid of a scanning probe microscope (5500, Agilent Technologies, Chandler, AZ, USA), using a reported procedure [33].

**X-ray Diffraction (XRD).** The crystallinity of the HDPE and HDPE-IS films was analyzed with a powder diffractometer (D500, Siemens, Munich, Germany) through a reported procedure [33].

**Differential Scanning Calorimetry (DSC).** A differential scanning calorimeter (Q20 V24.10 Build 122, TA Instruments, New Castle, DE, USA) was used to study the phase transitions of the HDPE and HDPE-IS films, using a reported protocol [33].

**Thermogravimetric Analysis (TGA).** The thermal degradation of the HDPE and HDPE-IS films was analyzed in a thermogravimetric analyzer (QA-50, TA Instruments, New Castle, DE, USA), using a reported protocol [33].

**Dynamic Mechanical Analysis (DMA).** The dynamic mechanical properties of the HDPE and HDPE-IS films were studied using a reported procedure [33], and a dynamic mechanical analyzer (Q800, TA Instruments, New Castle, DE, USA).

**Water Contact Angle Measurements.** The water contact angles were measured with the aid of a goniometer/drop shape analyzer (DSA100, Krüss, Hamburg, Germany), using a reported procedure [33].

**Antibiofilm Assay.** Petri dishes with Sabouraud agar containing chloramphenicol (HiMedia Laboratories LLC, Kelton, PA, USA) were employed to grow fresh yeast colonies (36 °C, 24 h). An inoculum (10^6^ CFU/mL) of the yeast colonies in tryptone soya broth (6 mL; HiMedia Laboratories LLC, Kelton, PA, USA) was prepared and incubated (36 °C, 24 h). HDPE films (1 × 1 cm) were sterilized (UV), inserted in a composition of peptone water (9 mL; HiMedia Laboratories LLC, Kelton, PA, USA) and tryptone soya broth inoculum (1 mL), and incubated for 96 h. The weakly adherent cells were removed using peptone water, and the films were inserted in flasks with peptone water (50 mL). After treatment under ultrasound (40 KHz, 10 min; Ultrasonic washer, USC-700, Unique Indústria e Comércio de Produtos Eletrônicos Ltda, Jardim Belo Horizonte, SP, Brazil), the solutions with the detached cells were diluted (10^−1^, 10^−2^, 10^−3^). These dilutions (20 µL) were plated in Petri dishes on Sabouraud agar containing chloramphenicol, and incubated (36 °C, 24 h). Finally, the number of CFU/cm^−2^ was determined and given logarithmically (log *M*, where *M* is the average value). Pure HDPE (film) was employed as a positive control [34].

**Minor Antibiofilm Concentration (MAC) Assay.** The protocols CLSI M27-A2 and CLSI M38-A were employed with minor modifications. Initially, the fresh yeast colonies were grown in Petri dishes on Sabouraud agar containing chloramphenicol (36 °C, 24 h; HiMedia Laboratories LLC, Kelton, PA, USA). After preparation of a 10^6^ CFU/mL yeast inoculum (100% transmittance for 0.9% saline and 90% transmittance for the 10^6^ CFU/mL yeast inoculum) in sterile saline (0.9%), aliquots (20 µL) were pipetted into 96-well microplates and complemented with Roswell Park Memorial Institute culture medium (180 µL; Gibco RPMI 1640, Thermo Fisher Scientific, Waltham, MA, USA). The HDPE films were cut in circles (5 mm diameter), sterilized (UV, 30 min), placed into the 96-well microplates, incubated (36 °C, 24 h), and then washed with sterile saline (0.9%, 1 mL; Sigma-Aldrich, Saint Louis, MO, USA). These films were placed into sterile 96-well microplates and 3-(4,5-dimethylthiazol-2-yl)-2,5-diphenyltetrazolium bromide (160 μL; Sigma-Aldrich, Saint Louis, MO, USA) was added to assess cell viability as a function of redox potential (3 h). The removal of the solution containing 3-(4,5-dimethylthiazol-2-yl)-2,5-diphenyltetrazolium bromide was followed by treatment (15 min) with isopropanol (160 µL; Sigma-Aldrich, Saint Louis, MO, USA). A microplate reader (EZ Read 400, Biochrom, Cambridge, United Kingdom) was used to determine the absorption intensities (570 and 690 nm) using 100 μL of each sample in isopropanol. The pure HDPE film in the mixture of yeast inoculum (20 µL) and RPMI (180 µL) was the positive control. For the negative control, RPMI (200 μL) was used [34]. The percentage of biofilm inhibition was determined through the formula: 100 − [(average assay absorbance)/(average absorbance of the positive control)] × 100.

**Biological Analysis on HDPE Films.** (A) In vitro cell culture: human Mesenchymal Stem Cells (hMSC, Lonza, Italy) at the fifth passage were employed to perform biological studies, using a reported procedure for the in vitro cell culture [33]. (B) Cell attachment—morphological analysis: A confocal microscope (TCS SP8, Leica Microsystems, Buccinasco, Milan, Italy) was employed to analyze cell-film interactions and spreading, using a fluorescent dye. In particular, HDPE films were cultured with 2 × 10^4^ cells (48 h; 37 °C); later, the non-attached cells were eliminated by careful washing with phosphate buffer solution (PBS; pH = 7.4, 0.01 M, Sigma-Aldrich, Milan, Italy), while the attached cells were treated with cell tracker green 5-chloromethylfluorescein diacetate (Life Technologies, Milan, Italy) in phenol red-free medium (37 °C; 30 min). The last step before the observation by CLSM consisted of washing with PBS and incubation in complete medium (1 h). (C) Biocompatibility test—attachment and proliferation: The biocompatibility test was performed on sterilized HDPE films (ethanol (4 h) and UV light (2 h)) equilibrated in Eagle’s alpha minimum essential medium (sterile-filtered, Sigma-Aldrich, Milan, Italy) overnight. Later, HDPE films with and without IS were seeded in triplicate with 1 × 10^4^ hMSCs and cultured (21 days). The effect of HDPE films on cell attachment and proliferation was quantitatively estimated by the Alamar blue assay (Life Technologies, Italy) at different time points. The results were reported as % of Alamar blue reduction (% AB reduction).

**Statistical Analysis.** One-way Analysis of Variance was employed with the multiple Dunnett comparison test, considering a significant difference for *P* < 0.05. The statistical analysis data were represented as mean ± standard deviation for n = 4 (antibiofilm, and MAC) or n = 3 (biocompatibility assay).

**Histopathological Evaluation in Pig Skin with HDPE Films.** The pig skin preparation, penetration and histopathological evaluation were performed following the reported procedures [33]. Ethic approval number: 04/2016 of the Animal Use Ethics Committee of the Federal Catarinense Institute - Campus Concórdia, Concórdia, SC, Brazil.

## 3. Results

HDPE-based biomaterials with IS additives were prepared by melt blending, followed by pressing into films. This resulted in the preparation of HDPE films with 0; 0.125; 0.250 and 0.500 wt% of either C_16_MImCl or C_16_MImMeS. In comparison to the rigid film of HDPE, increasing the content of IS made the films more flexible and less brittle.

**Figure 2:**
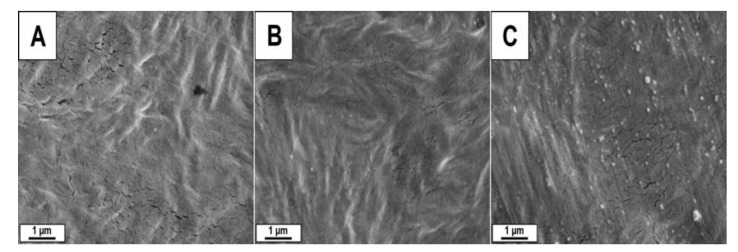
SEM micrographs of (**A**) HDPE, (**B**) HDPE.Cl.0500, and (**C**) HDPE.MeS.0500 (scale bar = 1 μm).

**Figure 3:**
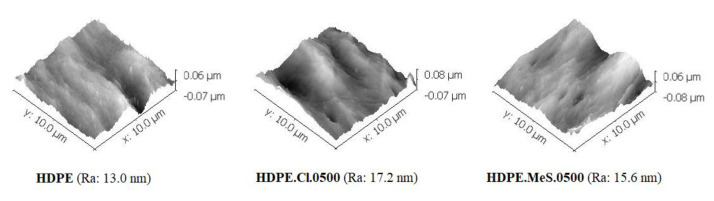
AFM images of HDPE, HDPE.Cl.0500, and HDPE.MeS.0500 (Ra = arithmetic mean roughness).

SEM investigations were performed to study the morphology of the HDPE-based films’ surfaces. The micrographs of HDPE, HDPE.Cl.0500 and HDPE.MeS.0500 (Figure 2) indicate that the IS incorporation did not have an expressive influence on the surface morphology of these biomaterials. This behavior is different compared to our previous work, in which the addition of C_16_MImCl and C_16_MImMeS in the PLLA matrix interfered with the morphology leading to the formation of superficial spheres and increasing the roughness of the final material [33]. This could be related to the difference in the procedures that were applied to obtain the films; solvent casting (PLLA) vs. pressure molding (HDPE), as well as the chemical interactions between the polymer and IS [35]. As such, the eventual effects of the ISs on the surface morphology of HDPE could have been eliminated during the transformation into films under heat and pressure, assuming the flat surface of the hydraulic press plates. This was further supported by the AFM images (Figure 3), where HDPE (roughness = 13.0 nm), HDPE.Cl.0500 (roughness = 17.2 nm) and HDPE.MeS.0500 (roughness = 15.6 nm) presented smooth surfaces. The somewhat higher roughness of HDPE.Cl.0500 was most likely related to its higher crystallinity, which will be presented in Table 1. This was less pronounced for HDPE.MeS.0500.

**Figure 4:**
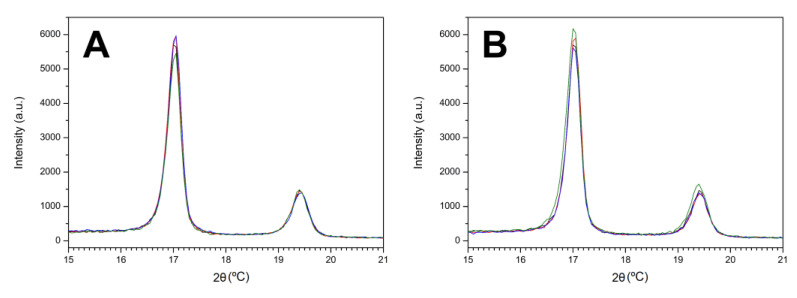
XRD diffractograms (a.u. = arbitrary units) within the 2θ range of (**A**) 15–25°, HDPE (black line), HDPE.Cl.0125 (red line), HDPE.Cl.0250 (blue line), and HDPE.Cl.0500 (green line) and (**B**) HDPE (black line), HDPE.MeS.0125 (red line), HDPE.MeS.0250 (blue line), and HDPE.MeS.0500 (green line).

Although the surface structure was not affected much by the presence of IS, the crystallinity of the HDPE-based biomaterials was studied by XRD (Figure 4). Independent of the IS (C_16_MImCl or C_16_MImMeS) or the IS content (0.125, 0.250 or 0.500 wt%), the type of HDPE crystallinity was not affected by obtaining crystalline HDPE.IS materials. All materials presented the typical HDPE peaks at 21.5° and 23.9°, which correspond to the (110) and (200) planes, respectively [36].

The thermal properties of the HDPE-based biomaterials were studied by DSC and TGA, and the results are given in Table 1. In general, the incorporation of IS in the contents of 0.125, 0.250 and 0.500 wt% led to subtle modifications in the thermal properties of the HDPE.IS biomaterials. The melting and crystallization temperatures (HDPE: 132.3 °C and 116.8 °C, respectively) varied within 1 °C. Compared to neat HDPE, HDPE.Cl.0500 showed increases of 5%, 4.4% and 5.6% in the melting enthalpy, the crystallization enthalpy and the crystallinity, respectively. The results also indicate that the ISs can be used as additives in the content range of 0.125–0.500 wt%, without modifying the thermal properties to a large extent. The same properties were studied with PLLA.IS biomaterials which showed an increase in thermal stability of 21 °C, whereas in this study the incorporation of IS into HDPE basically did not affect the thermal stability [33]. This effect may be related to the different intermolecular interactions between the ISs and the polymers. In PLLA.IS relatively strong non-covalent hydrogen bond interactions can take place whereas in HDPE.IS this is dependent on the weaker intermolecular van der Waals forces.

In Table 2 the storage and loss moduli and the stiffness results that were derived by DMA are summarized. Most of the HDPE.IS biomaterials showed similar storage moduli as HDPE, except for HDPE.Cl.0500, which showed lower values. For the loss moduli, the IS-containing HDPE films showed, in general, lower values although this did not follow a clear trend with increasing IS content, and HDPE.Cl.0500 presented a decrease of 24% in the value of HDPE. The lower storage and loss moduli and stiffness for HDPE.Cl.0500 could be related to its higher crystallinity as determined by DSC (Table 1). This could also explain the higher film roughness of HDPE.Cl.0500 (Figure 3). Interestingly, the biomaterials HDPE.Cl.0250, HDPE.MeS.0125 and HDPE.MeS.0500 demonstrated a better stiffness performance than the neat HDPE; the last one showed an increase of 35% in stiffness at 40 °C. No clear trend with the increase in IS load was observed indicating the non-linearity of the results. Generally, the dynamic-mechanical properties balance depended on the IS and its content, which was optimal for HDPE.MeS.0500. These results are in agreement with those obtained for PLLA.IS, which also generally demonstrated better values when C_16_MImMeS was employed [33].

(1) $$\mathrm{Xc}\;\left(\%\right)\;=\;\frac{\Delta \mathrm{Hm}}{\Delta H{}^{\circ}m\;\times \;\mathrm{Fp}}\;\times \;100\%$$

**Figure 5:**
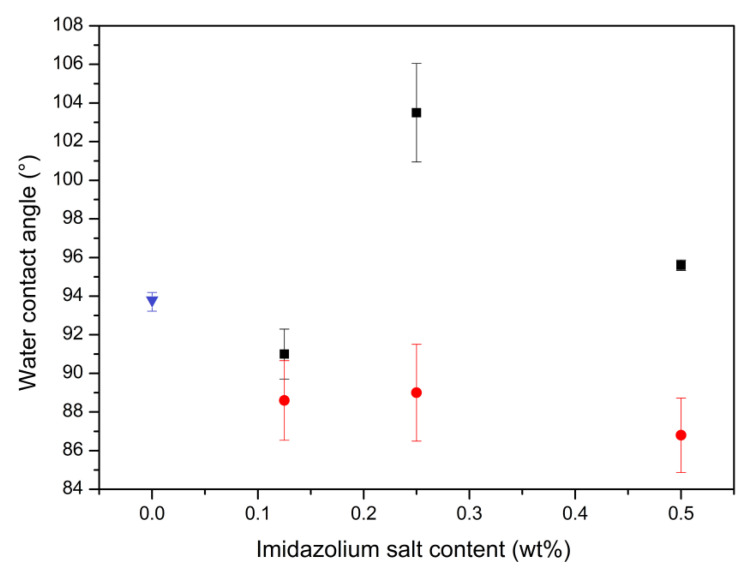
Water contact angles for HDPE films related to the IS content: HDPE (blue ▼), HDPE.Cl (black ◼), and HDPE.MeS (red ⬤).

To better understand the influence of IS dispersed in the HDPE-IS films regarding wettability properties, the water contact angle technique was applied (Figure 5). Although HDPE.Cl.0250 showed a higher water contact angle than HDPE, the other films with C_16_MImCl contents of 0.125 and 0.500 wt% only showed minor variations regarding the IS-free film. The increased hydrophobicity of HDPE.Cl.0250 suggests that C_16_MImCl was present at the surface and that its aliphatic part was preferentially oriented towards the water drop. In contrast, the HDPE films with C_16_MImMeS showed enhanced hydrophilicity according to the elevation of the IS load. The same effect was observed when 0.5 wt% of C_16_MImMeS was applied in PLLA [33]. This suggests again that the IS was present at the film surface and that the polar part (imidazolium cation ring and IS anion) was preferentially oriented towards the water drop.

Initially, the in vitro biofilm assay antibiofilm was performed to verify whether HDPE biomaterials with ISs exhibited an antibiofilm effect compared to HDPE (Figures S1–S3). In this test it was verified that in comparison with HDPE (without the ISs), all tested biomaterials (HDPE.Cl.0125, HDPE.Cl.0250, HDPE.Cl.0500, HDPE.MeS.0125, HDPE.MeS.0250 and HDPE.MeS.0500) showed antibiofilm activity against clinical isolates of *C. tropicalis* 72A, *C. parapsilosis* RL11 and RL20 and *C. albicans* CA04. Furthermore, HDPE films with C_16_MImCl (excluding HDPE.Cl.0250) also showed antibiofilm activity against *C. tropicalis* RL17.

**Figure 6:**
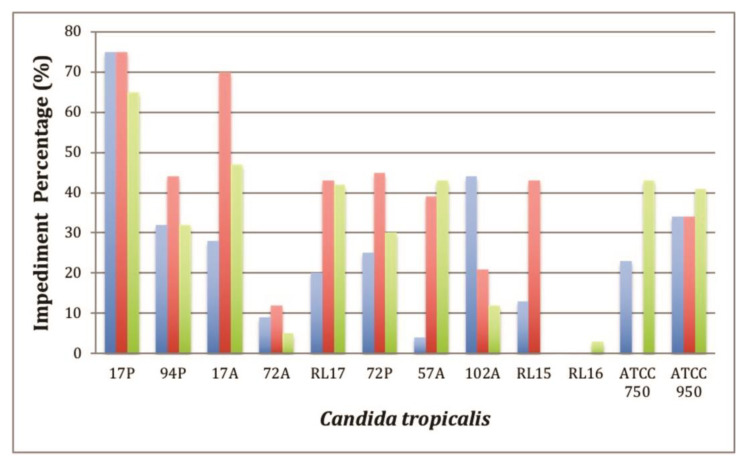
In vitro minor antibiofilm concentration assay: impediment percentage for HDPE.Cl.0125 (blue bars), HDPE.Cl.0250 (red bars), and HDPE.Cl.0500 (green bars).

**Figure 7:**
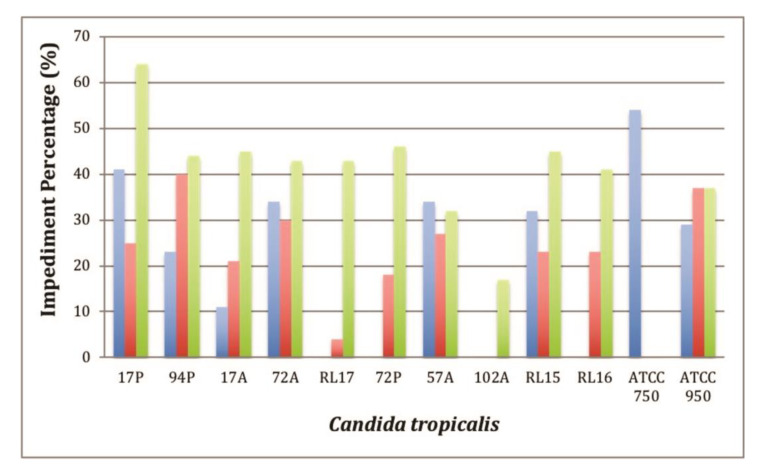
In vitro minor antibiofilm concentration assay: impediment percentage for HDPE.MeS.0125 (blue bars), HDPE.MeS.0250 (red bars), and HDPE.MeS.0500 (green bars).

Subsequently, the in vitro minor antibiofilm assay was performed to verify the percentage of prevention of biofilm formation on films of HDPE containing C_16_MImCl (HDPE.Cl.0125, HDPE.Cl.0250 and HDPE.Cl.0500) or C_16_MImMeS (HDPE.MeS.0125, HDPE.MeS.0250 and HDPE.MeS.0500), and the results are represented in Figure 6 and Figure 7, respectively. Those films were differentiated from the neat HDPE against 12 isolates of *C. tropicalis* that are well known to form biofilms [33,37]. The results of the statistical analysis are shown in Figures S4–S9. In general, the obtained results suggest that the presence of IS reduced the growth of biofilms compared to HDPE. The biofilm inhibition varied between 0–75% and 0–64% on the HDPE films containing C_16_MImCl and C_16_MImMeS, respectively. The inhibition percentage was dependent on the tested *C. tropicalis* isolate, which was possibly due to genetic mutations that made some isolates more resistant to the HDPE-IS biomaterials [38]. The best percentages of impediment of biofilm formation were obtained using HDPE.Cl.0125 and HDPE.Cl.0250 with 75% for *C. tropicalis* 17P. When HDPE.Cl.0500 was employed, a 65% impediment was obtained for *C. tropicalis* 17P and 47% for *C. tropicalis* 17A (Figure 6). The best percentages of impediment of biofilm formation using HDPE.MeS.0125 were 54% for *C. tropicalis* ATCC 750 and 41% for *C. tropicalis* 17P. HDPE.MeS.0250 demonstrated a 40% impediment for *C. tropicalis* 94P and 37% for *C. tropicalis* ATCC 950. In the case of HDPE.MeS.0500, a 64% impediment was obtained for *C. tropicalis* 17P and 46% for *C. tropicalis* 72P (Figure 7).

These results can be ascribed to the intrinsic antibiofilm property of IS, which was previously reported for C_16_MImCl in the pre-treatment of catheter surfaces [30], C_16_MImCl and C_16_MImMeS incorporated in PLA-based biomaterials [33], and imidazolium polymeric materials [27]. Now, this property was effectively transposed after their incorporation in HDPE. As HDPE alone is not an effective antibiofilm material, the ISs must be present on the biomaterial’s surface for this antibiofilm property to take place. As such, the prevention of biofilm formation was the result of a surface phenomenon due to the presence of IS at the HDPE surface. Even if IS would leach into the biological medium, its antibiofilm action will only take place when it is present on the surface of the biomaterial.

The results obtained with HDPE films with different contents of C_16_MImCl and C_16_MImMeS tested with *C. tropicalis* isolates showed that there is no direct relationship between the IS content used as an additive in HDPE and the percentage of prevention of formation of the biofilm (Figure 6 and Figure 7). Considering the effectiveness of the HDPE biomaterials in relation to the IS content compared to *C. tropicalis* isolates, it was possible to verify that at contents of 0.125 and 0.250 wt%, C_16_MImCl was more effective in preventing biofilm formation when compared to C_16_MImMeS. At the content of 0.500 wt%, C_16_MImMeS was more effective. In the case of PLA.IS, increasing the contents of the IS C_16_MImCl and C_16_MImMeS increased the percentage of impediment of biofilm formation [33]. The absence of this trend in the case of HDPE.IS suggests that other biomaterial properties impacted their determined antibiofilm potential including hydrophilicity and roughness [39,40].

**Figure 8:**
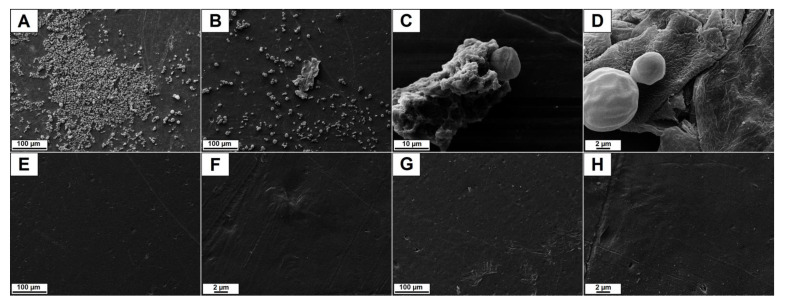
SEM micrographs of (**A**) HDPE (scale bar = 100 μm), (**B**) HDPE (scale bar = 100 μm), (**C**) HDPE (scale bar = 10 μm), (**D**) HDPE (scale bar = 2 μm), (**E**) HDPE.Cl.0500 (scale bar = 100 μm), (**F**) HDPE.Cl.0500 (scale bar = 2 μm), (**G**) HDPE.MeS.0500 (scale bar = 100 μm) and (**H**) HDPE.MeS.0500 (scale bar = 2 μm), after undergoing biofilm growth conditions.

Figure 8 shows SEM micrographs of HDPE samples. After 72 h of incubation with the clinical isolate *C. tropicalis* 72A (biofilm builder), the micrographs of HDPE (Figure 8A–D) show the formation of the biofilm with extracellular material and the cells at different stages of growth adhered to the HDPE film surface. In the cases of the films HDPE.Cl.0500 and HDPE.MeS.0500 (Figure 8E–H), the biofilm formation was prevented as no fungal and biofilm growth of *C. tropicalis* 72A was observed on the surfaces of these biomaterials. The results obtained indicate that, in the same way as PLLA.IS, both ISs were effective as anti-biofilm additives for HDPE.IS [33].

**Figure 9:**
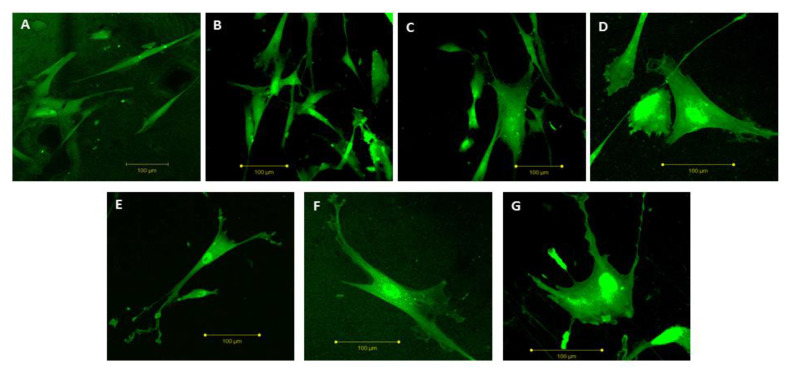
Confocal micrographs of hMSC grown on (**A**) HDPE, (**B**) HDPE.Cl.0125, (**C**) HDPE.Cl.0250, (**D**) HDPE.Cl.0500, (**E**) HDPE.MeS.0125, (**F**) HDPE.MeS.0250, and (**G**) HDPE.MeS.0500 (scale bar = 100 μm).

**Figure 10:**
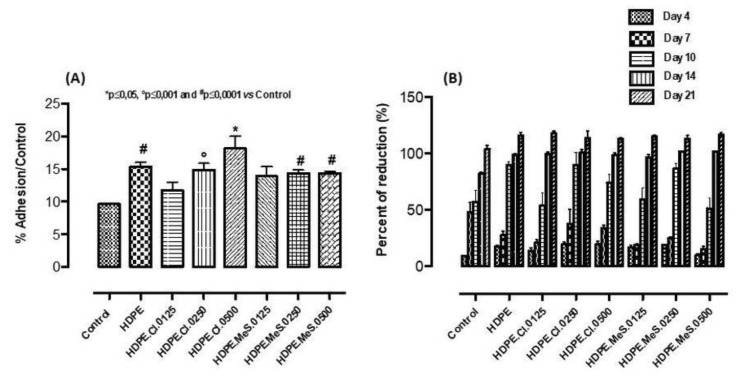
(**A**) hMSC adhesion after 48 h of incubation time and (**B**) hMSC proliferation at 4, 7, 10, 14 and 21 days of culture time.

The biocompatibility of a material is the principal parameter that governs the decision about the possibility to apply it in implants for human bodies. Such a biomaterial, when used in tissue engineering, should be non-toxic and biocompatible, without causing an intolerable degree of damage to that body [41]. In general, the in vitro cell-material interaction study is frequently used as an initial preliminary analysis of cell biocompatibility [42]. Herein, human mesenchymal stem cells (hMSC), generally used to evaluate the regeneration of mineralized extracellular matrix (ECM) in bone defects [43,44,45,46], were used for the in vitro testing of the biocompatibility of the HDPE-based materials with IS [43]. In particular, the effect of HDPE.IS biomaterials on the hMSC’s behavior was evaluated by cell adhesion, which is the first step involved in the biocompatibility process (Figure 9 and Figure 10A,B). Indeed, this cell attachment is the main stage to assess the influence of material surfaces on the hMSC behavior in the first hours of culture time. Both qualitative and quantitative analyses were performed with the aim to obtain information about the cell adhesion process. The morphological analysis (Figure 9) demonstrated a change in morphology with increasing IS contents. hMSC seeded on HDPE (without IS) showed a thin and elongated structure typical of fibroblast cells. Differently, the HDPE.IS biomaterials show a correlation between the presence of IS and the hMSC morphology. HDPE-based biomaterials with the ISs C_16_MImCl and C_16_MImMeS induced the stem cells to assume a polygonal structure, typical of osteoblast cells. This behavior was more pronounced for the biomaterials with higher IS contents (Figure 9C,D,F,G), which is highly favorable for bone repair processes. HDPE, **HDPE.Cl** and HDPE.MeS showed excellent values in the quantitative cell adhesion analysis which demonstrated good surface properties, promoting the extension of filopodia from the body cell and ensuring a stable cell attachment in the first 48 h of incubation (Figure 10A). For the C_16_MImCl-based biomaterials, the cell adhesion increased with an increasing IS content, showing a higher cell adhesion percentage for HDPE.Cl.0500 in comparison to HDPE. All HDPE.MeS samples showed values comparable to those obtained with **HDPE**. The confocal micrographs demonstrate that HDPE.Cl.0500 and HDPE.MeS.0500 improved the spreading of hMSC at the cell-material interface. Indeed, the cells are polygonal in shape, which is different from the elongated morphology observed for the substrates with lower IS amounts. These results were also obtained with PLLA.IS substrates in previous work as reported [33].

After the initial cell adhesion (Figure 10A), which is important for the next biocompatibility step, the cell proliferation after longer exposure times was studied (4, 7, 10, 14 and 21 days). This enables evaluating its continued cell development after initial adaptation to the biomaterial. In general, the HDPE materials, without and with IS, showed lower proliferation percentages than the control after 7 days (Figure 10B). Nevertheless, this was compensated for in all materials after 21 days; the cells became acquainted over time with their new environment. HDPE, HDPE.Cl.0250, HDPE.Cl.0500 and HDPE.MeS.0250 exceeded the proliferation percentages of the control after 10 days. The best proliferation results were achieved with the biomaterials containing 0.250 wt% of IS.

Finally, the results of the histopathological evaluation of skin of pig ear incubated with the HDPE films containing the ISs (Figure S10) showed no microscopic lesions.

## 4. Conclusions

In conclusion, the melt-blending of HDPE with the IS additives C_16_MImCl and C_16_MImMeS, and subsequent pressure molding, provided promising biomaterial films. Altogether, the ability of HDPE.IS to act effectively against the biofilm formation of Candida species, being biocompatible with hMSC, affording good cell adhesion and proliferation and being highly favorable for bone repair processes may open alternatives for the development of innovative medical devices.

## Figures and Tables

**Table 1:** Thermal Properties of HDPE Films.

Sample	T_m_^1^ [°C]	T_c_ ^2^ [°C]	ΔH_m_ ^3^ [J/g]	ΔH_c_ ^4^ [J/g]	X_c_ ^5^ [%]	T_5%_^6^ [°C]	T_10%_^7^ [°C]	T_50%_^8^ [°C]	Residue ^9^ [%]
HDPE	132.3	116.8	209.8	221.4	71.6	429.6	443.5	483.3	0.1
HDPE.Cl.0125 HDPE.MeS.0125	131.6	117.5	212.9	216.0	72.7	416.7	441.6	482.5	0.2
	132.0	117.2	212.8	207.9	72.7	431.0	445.1	483.0	0
HDPE.Cl.0250 HDPE.MeS.0250	131.7	117.6	205.7	223.9	70.4	400.8	429.3	473.1	0.7
	131.6	117.4	207.2	211.1	70.9	415.4	434.6	474.1	0
HDPE.Cl.0500 HDPE.MeS.0500	131.5	117.8	220.3	231.6	75.6	414.8	441.9	480.4	0.4
	131.8	117.3	206.1	210.1	70.7	412.2	432.6	481.4	0.7

^1^ Melting point obtained using DSC. ^2^ Crystallization temperature obtained using DSC. ^3^ Melting enthalpy obtained using DSC. ^4^ Crystallization enthalpy obtained using DSC. ^5^ Crystallinity obtained using DSC, and Equation (1), where ΔH^0^_m_ = 293 J/g for 100% crystalline HDPE [36], and Fp = polymer fraction. ^6^ Temperature at decomposition of 5 wt% obtained using TGA. ^7^ Temperature at decomposition of 10 wt% obtained using TGA. ^8^ Temperature at decomposition of 50 wt% obtained using TGA. ^9^ Residual weight at 550 °C obtained using TGA.

**Table 2:** Dynamic Mechanical Properties of HDPE Films.

Sample	G’-40 ^1^ [GPa]	G’40 ^2^ [GPa]	G’’-40 ^3^ [GPa]	G’’40 ^4^ [GPa]	S-40 ^5^ [kN/m]	S40 ^6^ [kN/m]	S90 ^7^ [kN/m]
HDPE	3.33	1.48	0.07	0.17	315.45	140.50	26.71
HDPE.Cl.0125 HDPE.MeS.0125	3.14	1.44	0.05	0.16	271.63	124.96	22.05
	3.17	1.40	0.06	0.16	349.31	154.66	28.11
HDPE.Cl.0250 HDPE.MeS.0250	3.28	1.52	0.07	0.16	346.19	160.34	29.83
	3.09	1.38	0.05	0.15	288.79	129.50	23.64
HDPE.Cl.0500	2.44	1.12	0.04	0.12	230.62	106.03	19.45
HDPE.MeS.0500	3.18	1.45	0.05	0.16	416.42	190.24	32.03

^1^ Stor*X*age modulus at −40 °C. ^2^ Storage modulus at 40 °C. ^3^ Loss modulus at −40 °C. ^4^ Loss modulus at 40 °C. ^5^ Stiffness at −40 °C. ^6^ Stiffness at 40 °C. ^7^ Stiffness at 90 °C.

## Data Availability

The data presented in this study are available on request from the corresponding author.

## Supplementary Materials

The following supporting information can be downloaded at: https://www.mdpi.com/article/10.3390/polym15051259/s1.
