# Supplementary material for: Imidazolium Salts for Candida spp. Antibiofilm High-Density Polyethylene-Based Biomaterials
Source: Polymers (Basel). 2023 Mar 1;15(5):1259. doi: 10.3390/polym15051259 (PMC10007465; doi:10.3390/polym15051259)
Supplement: Supplementary file 1 [file polymers-15-01259-s001.zip › Revision_SI_HDPE-IS Biomaterials_Proof.pdf]

Supporting Information

Imidazolium Salts for *Candida* spp. Antibiofilm High-Density Polyethylene-Based Biomaterials

Clarissa Martins Leal Schrekker[1], Yuri Clemente Andrade Sokolovicz[2], Maria Grazia Raucci[3], Claudio Alberto Martins Leal,[2] Luigi Ambrosio[3], Mário Lettieri Teixeira[4], Alexandre Meneghello Fuentefria[1,5,*], Henri Stephan Schrekker[2,*]

[1] Institute of Basic Health Sciences, Universidade Federal do Rio Grande do Sul (UFRGS), Rua Sarmento Leite 500, Porto Alegre, RS, 90050-170, Brazil; cla.schrekker@gmail.com (C.M.L.S.)

[2] Laboratory of Technological Processes and Catalysis, Institute of Chemistry, Universidade Federal do Rio Grande do Sul (UFRGS), Avenida Bento Gonçalves 9500, Porto Alegre, RS, 91501-970, Brazil; yurisokolovicz@gmail.com (Y.C.A.S.); camleal45@gmail.com (C.A.M.L.)

[3] Institute of Polymers, Composites and Biomaterials, National Research Council of Italy (IPCB-CNR), Viale John Fitzgerald Kennedy 54, Mostra d'Oltremare Padiglione 20, 80125 Naples, Italy; mariagrazia.raucci@cnr.it (M.G.R.); luigi.ambrosio@cnr.it (L.A.)

[4] Laboratory of Biochemistry and Toxicology, Instituto Federal Catarinense (IFC), Rodovia SC 283 – km 17, Concórdia, SC, 89703-720, Brazil; mario.teixeira@ifc.edu.br

[5] Faculty of Pharmacy, Universidade Federal do Rio Grande do Sul (UFRGS), Avenida Ipiranga 2752, Porto Alegre, RS, 90610-000, Brazil

*Correspondence: alexandre.fuentefria@ufrgs.br (A.M.F.); henri.schrekker@ufrgs.br (H.S.S.).

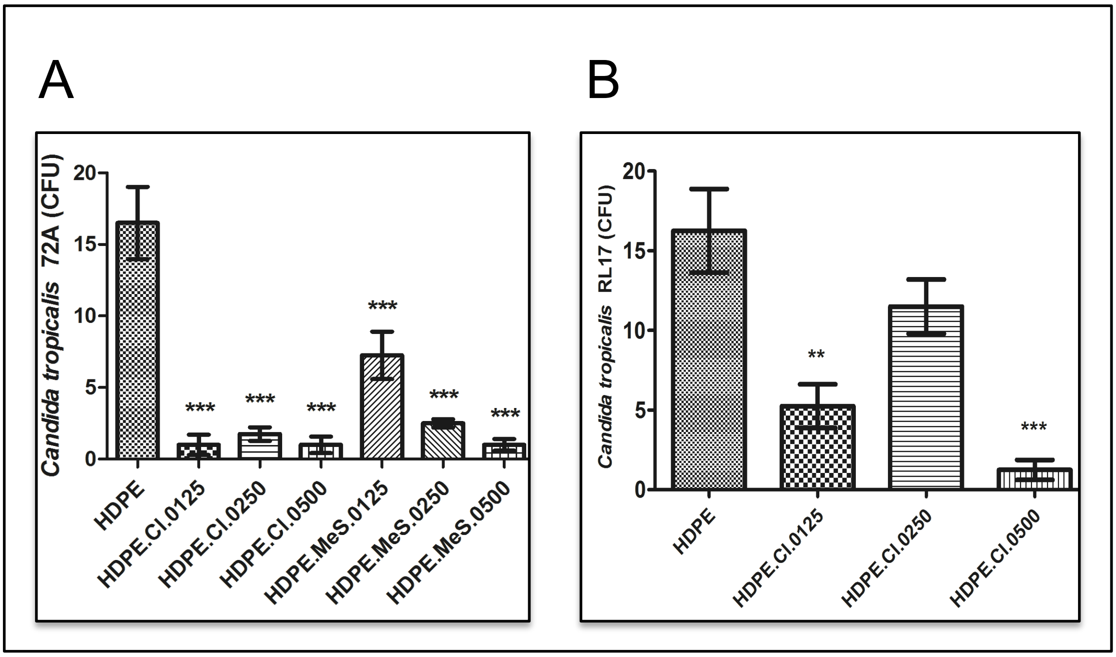

Figure S1. (**A**) Vertical bar graph of the biomaterial's antibiofilm activity, as determined by the antibiofilm assay (10^{-1} dilution) with HDPE, HDPE.Cl.0125, HDPE.Cl.0250, HDPE.Cl.0500, HDPE.MeS.0125, HDPE.MeS.0250 and HDPE.MeS.0500 against the *Candida tropicalis* 72A isolate. The data represent the mean ± standard deviation of 4 HDPE and 24 HDPE.IS samples. The P value was <0.0001, considered very significant for all HDPE.IS biomaterials. HDPE vs. HDPE.Cl.0125 *** P<0.001, HDPE vs. HDPE.Cl.0250 *** P<0.001, HDPE vs. HDPE.Cl.0500 *** P<0.001, HDPE vs. HDPE.MeS.0125 *** P<0.001, HDPE vs. HDPE.MeS.0250 *** P<0.001 and HDPE vs. HDPE.MeS.0500 *** P<0.001. (**B**) Vertical bar graph of the biomaterial's antibiofilm activity, as determined by the antibiofilm assay (10^{-1} dilution) with HDPE, HDPE.Cl.0125, HDPE.Cl.0250 and HDPE.Cl.0500, against the *C. tropicalis* RL17 isolate. The data represent the mean ± standard deviation of 4 HDPE and 12 HDPE.IS samples. The P value was 0.0003, considered significant for all HDPE.IS biomaterials. HDPE vs. HDPE.Cl.0125 ** P<0.01 and HDPE vs. HDPE.Cl.0500 *** P<0.001.

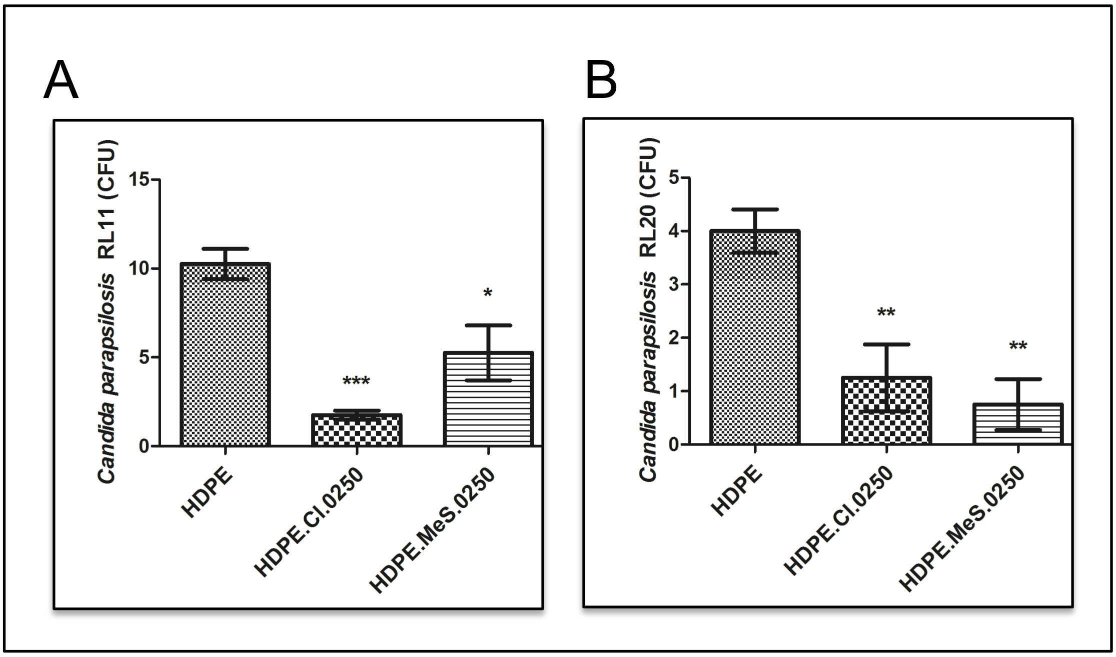

Figure S2. (**A**) Vertical bar graph of the biomaterial's antibiofilm activity, as determined by the antibiofilm assay (10^{-1} dilution) with HDPE, HDPE.Cl.0250 and HDPE.MeS.0250, against the *C. parapsilosis* RL11 isolate. The data represent the mean ± standard deviation of 4 HDPE and 8 HDPE.IS samples. The P value was 0.0008, considered significant for the HDPE.IS biomaterials. HDPE vs. HDPE.Cl.0250 *** $P<0.001$ and HDPE vs. HDPE.MeS.0250 * $P<0.05$. (**B**) Vertical bar graph of the biomaterial's antibiofilm activity, as determined by the antibiofilm assay (10^{-1} dilution) with HDPE, HDPE.Cl.0250 and HDPE.MeS.0250, against the *C. parapsilosis* RL20 isolate. The data represent the mean ± standard deviation of 4 HDPE and 8 HDPE.IS samples. The P value was 0.0032, considered very significant for all HDPE.IS biomaterials. HDPE vs. HDPE.Cl.0250 ** $P<0.01$ and HDPE vs. HDPE.MeS.0250 ** $P<0.01$.

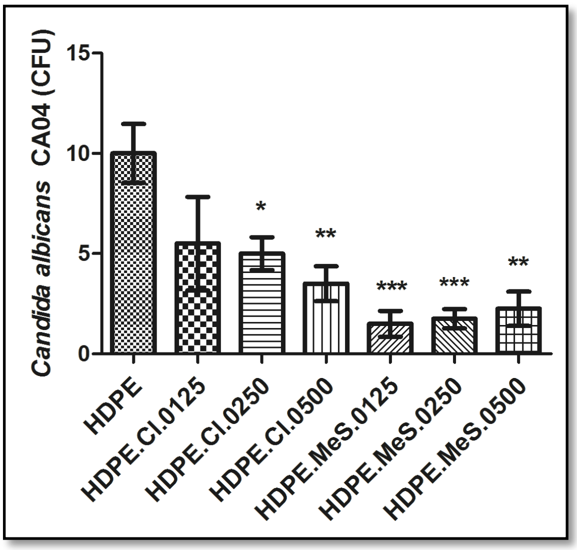

Figure S3. Vertical bar graph of the biomaterial's antibiofilm activity, as determined by the antibiofilm assay (10^{-1} dilution) with HDPE, HDPE.Cl.0125, HDPE.Cl.0250, HDPE.Cl.0500, HDPE.MeS.0125, HDPE.MeS.0250 and HDPE.MeS.0500, against the *C. albicans* CA04 isolate. The data represent the mean ± standard deviation of 4 HDPE and 24 HDPE.IS samples. The P value was 0.0009, considered significant for the HDPE.IS biomaterials. HDPE vs. HDPE.Cl.0250 * P<0.05, HDPE vs. HDPE.Cl.0500 ** P<0.01, HDPE vs. HDPE.MeS.0125 *** P<0.001, HDPE vs. HDPE.MeS.0250 *** P<0.001 and HDPE vs. HDPE.MeS.0500 ** P<0.01.

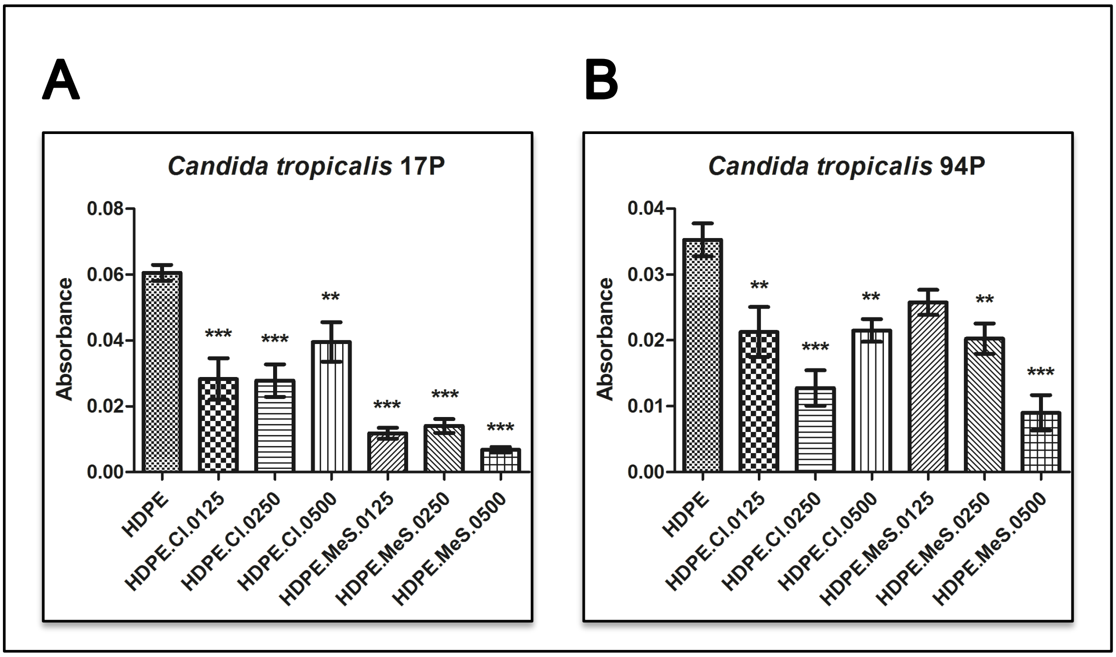

Figure S4. (**A**) Vertical bar graph of the biomaterial's biofilm impediment capacity, as determined by the absorbance value in the MAC assay with HDPE, HDPE.Cl.0125, HDPE.Cl.0250, HDPE.Cl.0500, HDPE.MeS.0125, HDPE.MeS.0250 and HDPE.MeS.0500, against the *C. tropicalis* 17P isolate. The data represent the mean ± standard deviation of 4 HDPE and 24 HDPE.IS samples. The P value was <0.0001, considered significant for the HDPE.IS biomaterials. HDPE vs. HDPE.Cl.0125 *** P<0.001, HDPE vs. HDPE.Cl.0250 *** P<0,001, HDPE vs. HDPE.Cl.0500 ** P<0.01, HDPE vs. HDPE.MeS.0125 *** P<0.001, HDPE vs. HDPE.MeS.0250 *** P<0.001 and HDPE vs. HDPE.MeS.0500 *** P<0.001. (**B**) Vertical bar graph of the biomaterial's biofilm impediment capacity, as determined by the absorbance value in the MAC assay with HDPE, HDPE.Cl.0125, HDPE.Cl.0250, HDPE.Cl.0500, HDPE.MeS.0125, HDPE.MeS.0250 and HDPE.MeS.0500, against the *C. tropicalis* 94P isolate. The data represent the mean ± standard deviation of 4 HDPE and 24 HDPE.IS samples. The P value was <0.0001, considered significant for the HDPE.IS biomaterials. HDPE vs. HDPE.Cl.0125 ** P<0.01, HDPE vs. HDPE.Cl.0250 *** P<0.001, HDPE vs. HDPE.Cl.0500 ** P<0.01, HDPE vs. HDPE.MeS.0250 ** P<0.01 and HDPE vs. HDPE.MeS.0500 *** P<0.001.

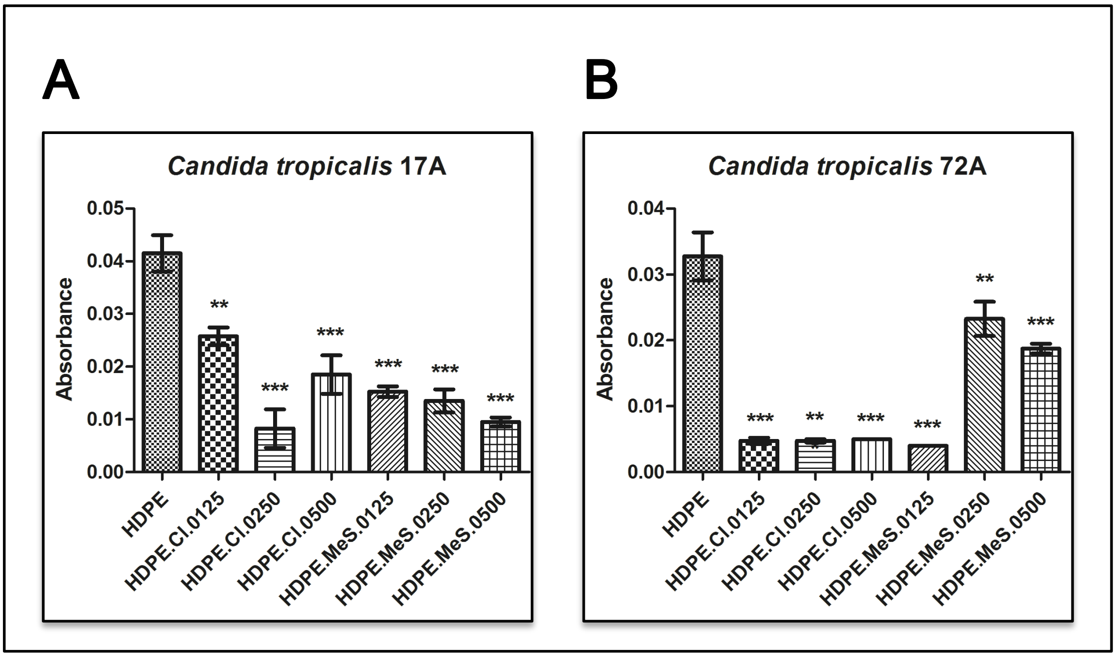

Figure S5. (**A**) Vertical bar graph of the biomaterial's biofilm impediment capacity, as determined by the absorbance value in the MAC assay with HDPE, HDPE.Cl.0125, HDPE.Cl.0250, HDPE.Cl.0500, HDPE.MeS.0125, HDPE.MeS.0250 and HDPE.MeS.0500, against the *C. tropicalis* 17A isolate. The data represent the mean ± standard deviation of 4 HDPE and 24 HDPE.IS samples. The P value was <0.0001, considered significant for the HDPE.IS biomaterials. HDPE vs. HDPE.Cl.0125 ** P<0.01, HDPE vs. HDPE.Cl.0250 *** P<0.001, HDPE vs. HDPE.Cl.0500 *** P<0.001, HDPE vs. HDPE.MeS.0125 *** P<0.001, HDPE vs. HDPE.MeS.0250 *** P<0.001 and HDPE vs. HDPE.MeS.0500 *** P<0.001. (**B**) Vertical bar graph of the biomaterial's biofilm impediment capacity, as determined by the absorbance value in the MAC assay with HDPE, HDPE.Cl.0125, HDPE.Cl.0250, HDPE.Cl.0500, HDPE.MeS.0125, HDPE.MeS.0250 and HDPE.MeS.0500, against the *C.* 72A isolate. The data represent the mean ± standard deviation of 4 HDPE and 28 HDPE.IS samples. The P value was <0.0001, considered significant for the HDPE.IS biomaterials. HDPE vs. HDPE.Cl.0125 *** P<0.001, HDPE vs. HDPE.Cl.0250 ** P<0.01, HDPE vs. HDPE.Cl.0500 *** P<0.001, HDPE vs. HDPE.MeS.0125 *** P<0.001, HDPE vs. HDPE.MeS.0250 ** P<0.01 and HDPE vs. HDPE.MeS.0500 *** P<0.001.

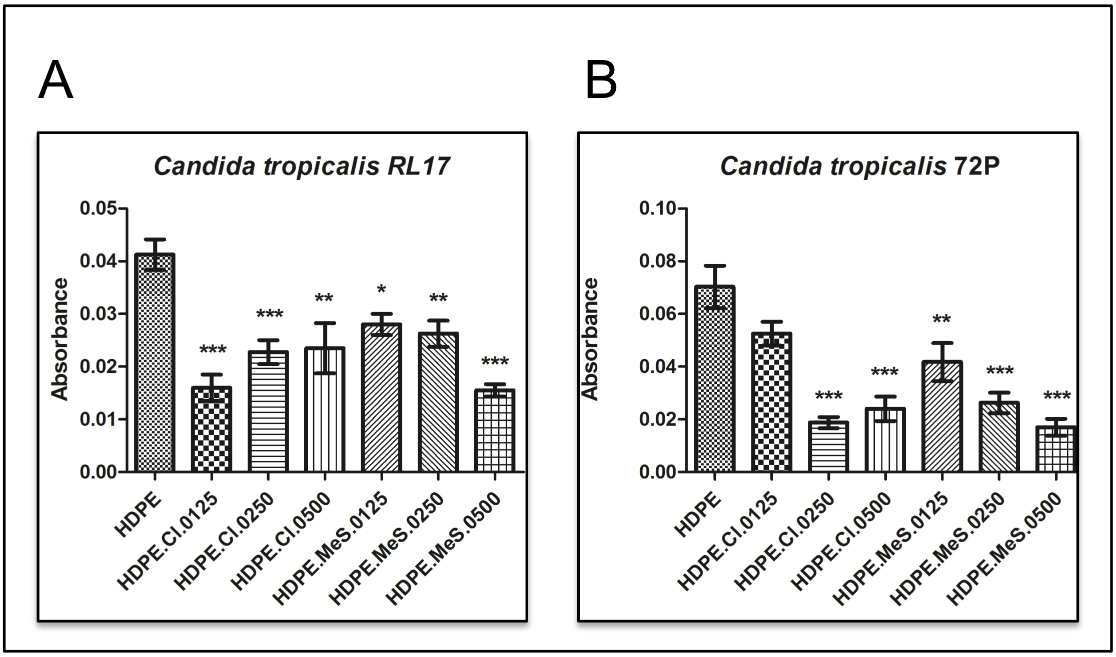

Figure S6. (**A**) Vertical bar graph of the biomaterial's biofilm impediment capacity, as determined by the absorbance value in the MAC assay with HDPE, HDPE.Cl.0125, HDPE.Cl.0250, HDPE.Cl.0500, HDPE.MeS.0125, HDPE.MeS.0250 and HDPE.MeS.0500, against the *C. tropicalis* 17A isolate. The data represent the mean ± standard deviation of 4 HDPE and 24 HDPE.IS samples. The P value was <0.0001, considered significant for the HDPE.IS biomaterials. HDPE vs. HDPE.Cl.0125 ** P<0.01, HDPE vs. HDPE.Cl.0250 *** P<0.001, HDPE vs. HDPE.Cl.0500 *** P<0.001, HDPE vs. HDPE.MeS.0125 *** P<0.001, HDPE vs. HDPE.MeS.0250 *** P<0.001 and HDPE vs. HDPE.MeS.0500 *** P<0.001. (**B**) Vertical bar graph of the biomaterial's biofilm impediment capacity, as determined by the absorbance value in the MAC assay with HDPE, HDPE.Cl.0125, HDPE.Cl.0250, HDPE.Cl.0500, HDPE.MeS.0125, HDPE.MeS.0250 and HDPE.MeS.0500, against the *C.* 72A isolate. The data represent the mean ± standard deviation of 4 HDPE and 28 HDPE.IS samples. The P value was <0.0001, considered significant for the HDPE.IS biomaterials. HDPE vs. HDPE.Cl.0125 *** P<0.001, HDPE vs. HDPE.Cl.0250 ** P<0.01, HDPE vs. HDPE.Cl.0500 *** P<0.001, HDPE vs. HDPE.MeS.0125 *** P<0.001, HDPE vs. HDPE.MeS.0250 ** P<0.01 and HDPE vs. HDPE.MeS.0500 *** P<0.001.

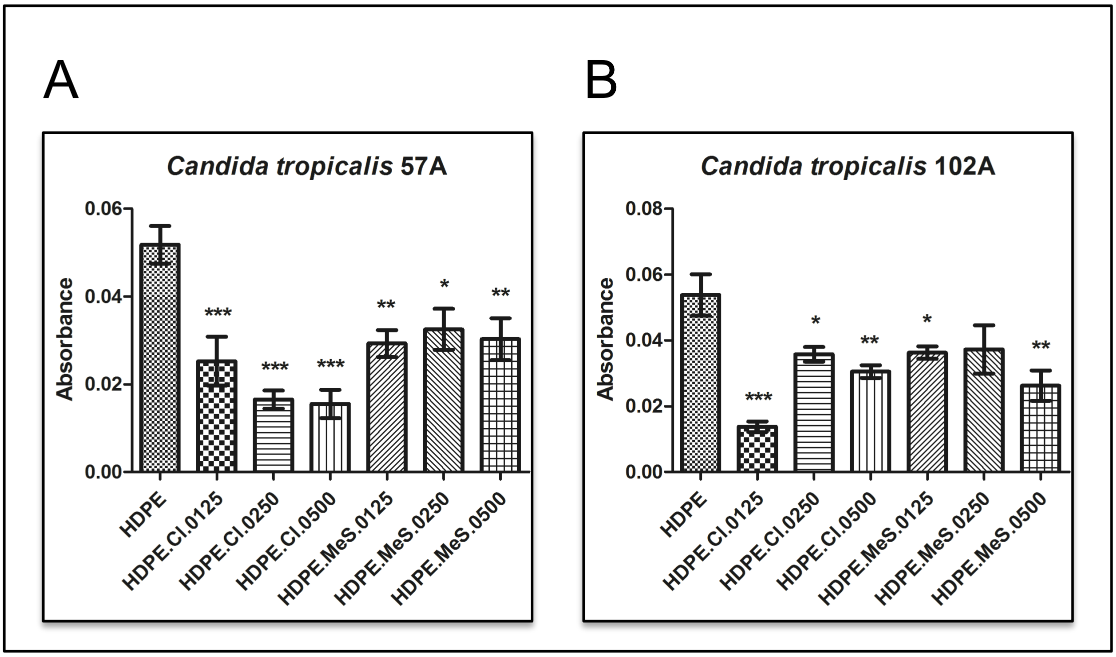

Figure S7. (**A**) Vertical bar graph of the biomaterial's biofilm impediment capacity, as determined by the absorbance value in the MAC assay with HDPE, HDPE.Cl.0125, HDPE.Cl.0250, HDPE.Cl.0500, HDPE.MeS.0125, HDPE.MeS.0250 and HDPE.MeS.0500, against the *C. tropicalis* 57A isolate. The data represent the mean ± standard deviation of 4 HDPE and 28 HDPE.IS samples. The P value was <0.0001, considered significant for the HDPE.IS biomaterials. HDPE vs. HDPE.Cl.0125 *** P<0.001, HDPE vs. HDPE.Cl.0250 *** P<0.001, HDPE vs. HDPE.Cl.0500 *** P<0.001, HDPE vs. HDPE.MeS.0125 ** P<0.01, HDPE vs. HDPE.MeS.0250 * P<0.05 and HDPE vs. HDPE.MeS.0500 ** P<0.01. (**B**) Vertical bar graph of the biomaterial's biofilm impediment capacity, as determined by the absorbance value in the MAC assay with HDPE, HDPE.Cl.0125, HDPE.Cl.0250, HDPE.Cl.0500, HDPE.MeS.0125, HDPE.MeS.0250 and HDPE.MeS.0500, against the *C. tropicalis* 102A isolate. The data represent the mean ± standard deviation of 4 HDPE and 28 HDPE.IS samples. The P value was 0.0001, considered significant for the HDPE.IS biomaterials. HDPE vs. HDPE.Cl.0125 *** P<0.001, HDPE vs. HDPE.Cl.0250 * P<0.05, HDPE vs. HDPE.Cl.0500 ** P<0.01, HDPE vs. HDPE.MeS.0125 * P<0.05 and HDPE vs. HDPE.MeS.0500 ** P<0.01.

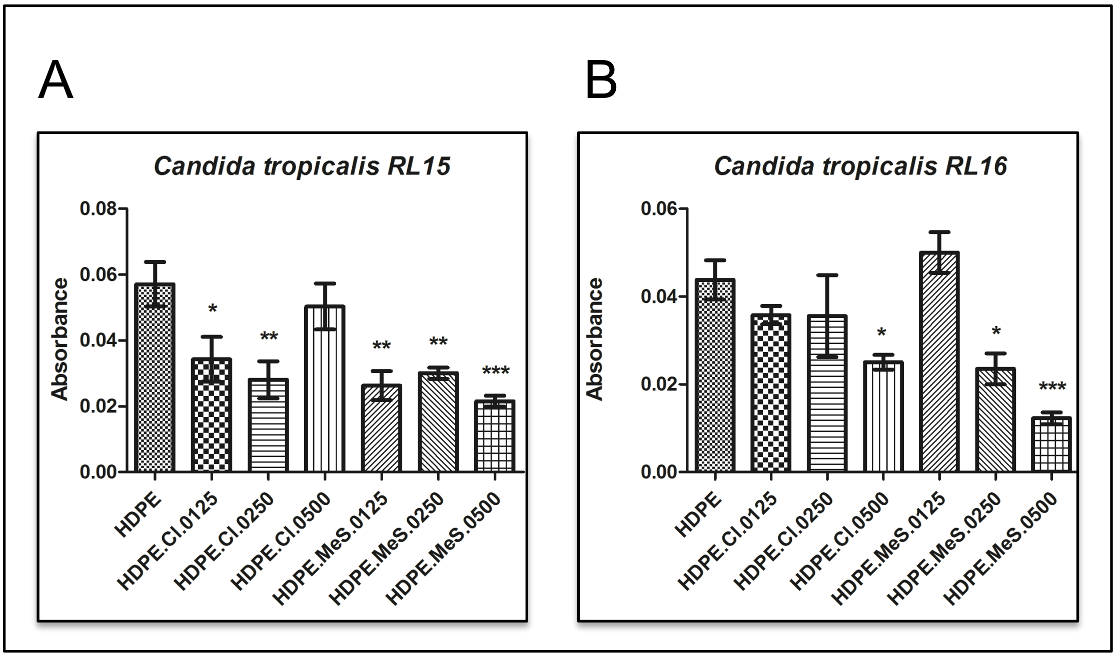

Figure S8. (**A**) Vertical bar graph of the biomaterial's biofilm impediment capacity, as determined by the absorbance value in the MAC assay with HDPE, HDPE.Cl.0125, HDPE.Cl.0250, HDPE.Cl.0500, HDPE.MeS.0125, HDPE.MeS.0250 and HDPE.MeS.0500, against the *C. tropicalis* RL15 isolate. The data represent the mean ± standard deviation of 4 HDPE and 24 HDPE.IS samples. The P value was 0.0007, considered significant for the HDPE.IS biomaterials. HDPE vs. HDPE.Cl.0125 * $P<0.05$, HDPE vs. HDPE.Cl.0250 ** $P<0.01$, HDPE vs. HDPE.MeS.0125 ** $P<0.01$, HDPE vs. HDPE.MeS.0250 ** $P<0.01$ and HDPE vs. HDPE.MeS.0500 *** $P<0.001$. (**B**) Vertical bar graph of the biomaterial's biofilm impediment capacity, as determined by the absorbance value in the MAC assay with HDPE, HDPE.Cl.0125, HDPE.Cl.0250, HDPE.Cl.0500, HDPE.MeS.0125, HDPE.MeS.0250 and HDPE.MeS.0500, against the *C. tropicalis* RL16 isolate. The data represent the mean ± standard deviation of 4 HDPE and 24 HDPE.IS samples. The P value was 0.0002, considered significant for the HDPE.IS biomaterials. HDPE vs. HDPE.Cl.0500 * $P<0.05$, HDPE vs. HDPE.MeS.0250 * $P<0.05$ and HDPE vs. HDPE.MeS.0500 *** $P<0.001$.

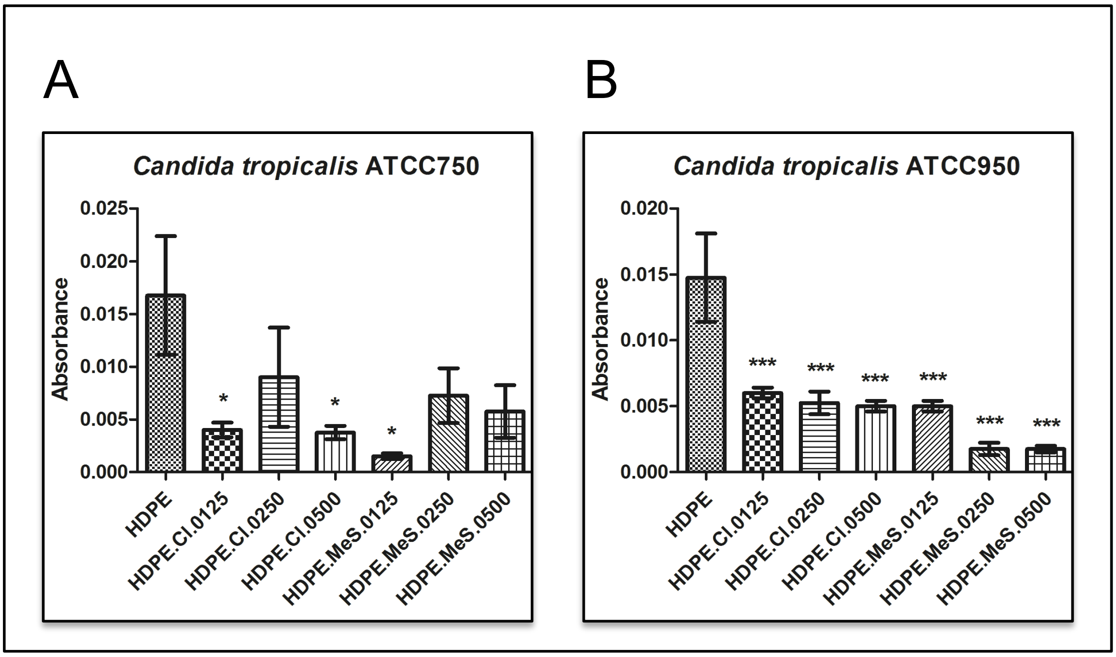

Figure S9. (**A**) Vertical bar graph of the biomaterial's biofilm impediment capacity, as determined by the absorbance value in the MAC assay with HDPE, HDPE.Cl.0125, HDPE.Cl.0250, HDPE.Cl.0500, HDPE.MeS.0125, HDPE.MeS.0250 and HDPE.MeS.0500, against the *C. tropicalis* ATCC750 isolate. The data represent the mean ± standard deviation of 4 HDPE and 24 HDPE.IS samples. The P value was 0.0491, considered significant for the HDPE.IS biomaterials. HDPE vs. HDPE.Cl.0125 * P<0.05, HDPE vs. HDPE.Cl.0500 * P<0.05 and HDPE vs. HDPE.MeS.0125 * P<0.05. (**B**) Vertical bar graph of the biomaterial's biofilm impediment capacity, as determined by the absorbance value in the MAC assay with HDPE, HDPE.Cl.0125, HDPE.Cl.0250, HDPE.Cl.0500, HDPE.MeS.0125, HDPE.MeS.0250 and HDPE.MeS.0500, against the *C. tropicalis* ATCC950 isolate. The data represent the mean ± standard deviation of 4 HDPE and 24 HDPE.IS samples. The P value was <0.0001, considered significant for the HDPE.IS biomaterials. HDPE vs. HDPE.Cl.0125 *** P<0.001, HDPE vs. HDPE.Cl.0250 *** P<0.001, HDPE vs. HDPE.Cl.0500 *** P<0.001, HDPE vs. HDPE.MeS.0125 *** P<0.001, HDPE vs. HDPE.MeS.0250 *** P<0.001 and HDPE vs. HDPE.MeS.0500 *** P<0.001.

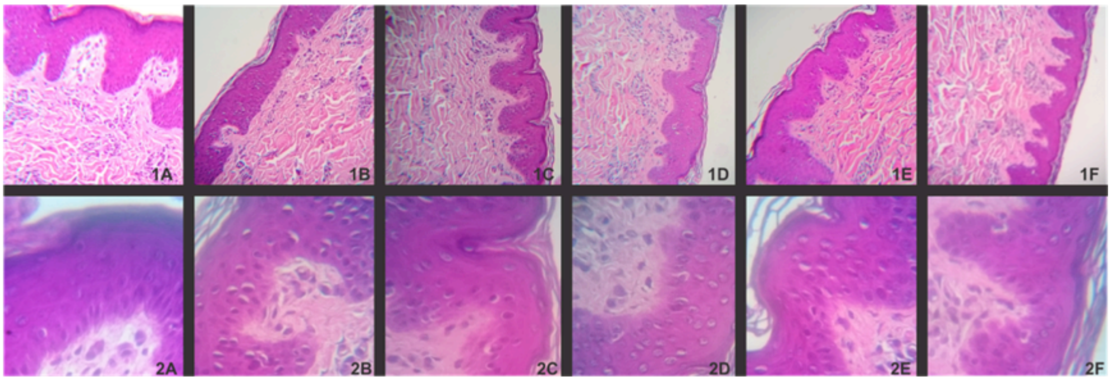

Figure S10. Histopathological evaluation of pig ear skin in contact with HDPE films that contain IS (C_{16}MImCl or C_{16}MImMeS) and treated with PBS pH 7.0 buffer solution (negative control in increments of 100 and 400 times in magnitude): 100-Fold magnification of pig ear epithelial cells treated with (**1A**) PBS pH 7.0 buffer; (**1B**) HDPE; (**1C**) HDPE.Cl.0250; (**1D**) HDPE.Cl.0500; (**1E**) HDPE.MeS.0250; and (**1F**) HDPE.MeS.0500. 400-Fold magnification of pig ear epithelial cells treated with (**2A**) PBS pH 7.0 buffer; (**2B**) HDPE; (**2C**) HDPE.Cl.0250; (**2D**) HDPE.Cl.0500; (**2E**) HDPE.MeS.0250; and (**2F**) HDPE.MeS.0500.
